# PCSK9 Prosegment Chimera as Novel Inhibitors of LDLR Degradation

**DOI:** 10.1371/journal.pone.0072113

**Published:** 2013-08-12

**Authors:** Yascara Grisel Luna Saavedra, Jianbing Zhang, Nabil G. Seidah

**Affiliations:** 1 Laboratory of Biochemical Neuroendocrinology, Clinical Research Institute of Montreal, affiliated to the Université de Montréal, Montréal, Québec, Canada; 2 Institute for Biological Sciences, National Research Council Canada, Ottawa, Ontario, Canada; Cardiological Center, Italy

## Abstract

The proprotein convertase PCSK9, a target for the treatment of hypercholesterolemia, is a negative regulator of the LDL receptor (LDLR) leading to its degradation in endosomes/lysosomes and up-regulation of plasma LDL-cholesterol levels. The proprotein convertases, a family of nine secretory serine proteases, are first synthesized as inactive zymogens. Except for PCSK9, all other convertases are activated following the autocatalytic excision of their inhibitory N-terminal prosegment. PCSK9 is unique since the mature enzyme exhibits a cleaved prosegment complexed with the catalytic subunit and has no protease activity towards other substrates. Similar to other convertases, we hypothesized that the *in trans* presence of the PCSK9 prosegment would interfere with PCSK9's activity on the LDLR. Since the prosegment cannot be secreted alone, we engineered a chimeric protein using the Fc-region of human IgG1 fused to the PCSK9 prosegment. The expression of such Fcpro-fusion protein in HEK293 and HepG2 cells resulted in a secreted protein that binds PCSK9 and markedly inhibits its activity on the LDLR. This was observed by either intracellular co-expression of PCSK9 and Fcpro or by an extracellular *in vitro* co-incubation of Fcpro with PCSK9. Structure-function studies revealed that the inhibitory function of Fcpro does not require the acidic N-terminal stretch (residues 31–58) nor the C-terminal Gln**_152_** of the prosegment. Fcpro likely interacts with the prosegment and/or catalytic subunit of the prosegment≡PCSK9 complex thereby allosterically modulating its function. Our data suggest a novel strategic approach for the design and isolation of PCSK9 inhibitors.

## Introduction

The mammalian proprotein convertases (PCs) [1] are members of a secretory serine protease family composed of nine members related to bacterial subtilisin and yeast kexin. Seven of these (PC1/3, PC2, Furin, PC4, PC5/6, PACE4 and PC7) exhibit homology of their catalytic domain to that of yeast kexin, and are known to cleave after basic residues. The eighth member, SKI-1/S1P, shows homology to bacterial pyrolysin and cleave after non-basic residues. Finally, the last member, PCSK9, shows homology to fungal proteinase K and cleaves itself once in the endoplasmic reticulum at the (V/I)FAQ↓ motif. Like many other proteases, these convertases are synthesized as inactive zymogens. Their prosegment located at their N-terminus is implicated in the productive folding of the enzyme and in its stabilization as an inactive form, like a natural inhibitor, until one or more cleavages occur followed by the release of the active enzyme dissociated from its prosegment [2].

Five PCs regulate sterols and/or lipid metabolism (Furin, PC5/6, PACE4, SKI-1/S1P and PCSK9). Among these, the gene coding for convertase PCSK9 [3] was discovered to be the third locus implicated in Familial Hypercholesterolemia (FH3) [4]. Since 2003, *in vitro* and *in vivo* studies unraveled the physiological roles of PCSK9 in the regulation of the cholesterol and fatty acid metabolism. PCSK9 is highly expressed in liver hepatocytes and is first synthesized as a pre-proprotein convertase. During its passage through the secretory pathway and at the level of the endoplasmic reticulum (ER), the zymogen gets autocatalytically cleaved at VFAQ_152_↓SIP separating its prosegment from the catalytic domain. The cleaved C-terminus of the prosegment then occupies the catalytic pocket of the enzyme and blocks access to other exogenous substrates [5]–[7]. The complex prosegment≡PCSK9 (herein abbreviated pPCSK9) then exits the ER and reaches the Golgi apparatus leading to its rapid secretion into the medium [3] or in plasma. Through its catalytic domain, mature PCSK9 binds the EGF-A domain of the LDL receptor (LDLR) [8] both intracellularly in the TGN [9] and at the cell surface [10]. Once the non-covalent complex pPCSK9≡LDLR is formed, it gets internalized by endocytosis and directed to degradation in the acidic compartments of endosomes/lysosomes [11], [12] by an as yet unknown mechanism. Thus, PCSK9 acts as a negative regulator of the cellular LDLR protein by preventing its recycling to the cell surface. This down-regulation and the subsequent accumulation of LDL particles (LDLR natural ligand) in plasma lead to hypercholesterolemia. LDL particles being atherogenic, they obstruct the luminal side of vessels resulting in vascular complications such as atherosclerosis, stroke and premature heart attacks [13].

Since the worldwide discovery of individuals harboring natural mutations of PCSK9, clinical studies have established a causative association between “gain of function” (GOF) mutations with hypercholesterolemia [4] and “loss of function” (LOF) mutations with hypocholesterolemia [14]. Moreover, the identification of two seemingly healthy individuals carrying LOF mutations in both alleles, which lead to a complete absence of circulating PCSK9 and correlating with very low plasma LDL-cholesterol levels was a major breakthrough that encouraged the scientific community to develop PCSK9 inhibitors as a novel treatment of hypercholesterolemia [1].

As for all members of the proprotein convertase family, the zymogen of PCSK9 has a prosegment located at the N-terminus followed by a subtilisin-like catalytic domain and a C-terminal segment. The prosegment itself serves as intramolecular chaperone ensuring the correct folding of the enzyme during the maturation process. Consistently, such zymogens undergo an intramolecular cleavage between their prosegment and their catalytic domain followed, in most cases, by a second cleavage within the prosegment. This allows the convertases to get rid of their inhibitory prosegment and the generation of an active protease. One of the peculiarities of PCSK9 compared to other convertases is its inability to get rid of its prosegment. In fact, immediately after the first intramolecular cleavage in the ER, the C-terminal extremity of the prosegment binds tightly to the catalytic pocket. As suggested by X-ray structure studies [5]–[7], the prosegment acts as a “specific inhibitor” of PCSK9 preventing any further enzymatic activity. Since we previously demonstrated that the prosegments of the PCs can act as potent inhibitors of these convertases both *in vitro* and *ex vivo* in cell lines [15], we hypothesized that the PCSK9 prosegment may also function as an effective inhibitor blocking the activity of the pPCSK9 on LDLR degradation. If true, ultimately this would represent a novel strategy to inhibit PCSK9 function and hence increase cellular LDLR levels.

In the present study we generated a recombinant chimeric protein called Fcpro by taking advantage of the growing class of human therapeutics consisting in the use of the constant Fc domain of the human immunoglobulin G (hIgG1) to build stable recombinant fusion proteins. Herein, we provide evidence that when fused to an Fc fragment such chimeric PCSK9 prosegment (Fcpro) can be well expressed and secreted. We also show that the recombinant Fcpro protein is able to directly bind PCSK9 and block its activity towards the degradation of the LDLR by an intracellular manner as shown by our co-expression experiments or by an extracellular route when both proteins are co-incubated. The interaction of recombinant Fcpro with wild type PCSK9 or its gain-of-function mutants resulted in a recovery of the cellular LDLR levels.

## Materials and Methods

### Plasmids and reagents

Human PCSK9 and its mutant cDNAs (L455X, CHRD, D374Y, Δ33-58, Δ33-58 D374Y) were cloned into pIRES2-EGFP (Clontech, Mountain view CA) as described [3], [12]. Human HepG2/shPCSK9 cells essentially lacking endogenous PCSK9 [16] and HEK293 cells (American Type Culture Collection) were cultivated in Dulbecco's modified Eagle's medium (DMEM; Gibco) supplemented with 10% fetal bovine serum (FBS; Wisent). Puromycin (2 µg/mL; Invitrogen) was added only to HepG2/shPCSK9 cells as a selection antibiotic. Lipoprotein deficient serum (LPDS) was from Biomedical technologies.

### cDNA constructs

The construct pIRES2-EFGP-human PCSK9-V5 (C-terminal V5-tag) [3] was used as a template to generate cDNAs coding for human PCSK9 mutants. Two-step PCRs were used to introduce deletion mutants as described previously [11]. All constructs contain a V5 tag at the C-terminus. The cDNA coding for the Fcpro recombinant protein (*alias* pTT5-Fc1_hPSM) was generated as described [17]. Briefly, a vector pTT5-hFc2 was first constructed to facilitate the insertion of human prosegment of (pro *alias* hPSM) PCSK9. Mutations were made at the C-terminus of Fc of human IgG1 of pTT5-hFc11 vector to introduce restriction sites of NsiI and HindIII without changing the amino acid sequence of Fc, generating vector pTT5-hFc2. DNA coding for the prosegment of PCSK9 (amino acid, aa 31–152) was synthesized at GeneArt, to which hinge region of human IgG1 was added at the N-terminus and NsiI and HindIII restriction sites were introduced using standard PCR technique. The fragment was subsequently cloned into pTT5-hFc2, which resulted in pTT5-Fc1_hPSM (Fcpro), an expression vector of a fusion protein consisting of Fc of human IgG1, hinge range of human IgG1 and PCSK9 prosegment (pro). This arrangement is made based on the consideration that the C-terminus of human prosegment should be left free to allow its binding to human PCSK9. Human Fc was included to extend the likely short serum half life of human PCSK9's prosegment, and the hinge range was included to allow free movement of the prosegment. All constructs were confirmed by DNA sequencing.

### Cell culture and transfections

HepG2/shPCSK9 cells were seeded at 1×10^5^ cells/well in a 12 well microplate (Greiner Bio-One). After 24 h, the cells were transfected with 1 µg cDNAs using Fugene HD (Roche Applied Science), and 24 h post-transfection, the cells were washed and then incubated with fresh DMEM medium without serum for an additional 24 h before recovering media and cells.

### Preparation of conditioned media

HEK293 or HepG2/shPCSK9 cells were seeded at 2×10^6^ cells/100 mm petri dish coated with poly-L-Lysine (Invitrogen) and were transfected with a total of 4 µg of cDNA using Effectene (Qiagen) or Fugene HD (Roche) respectively. At 24 h post-transfection, the cells were washed and incubated with serum free media. Conditioned media were recovered 72 h post-transfection. The spent media were then concentrated on an Amicon Ultra-15 centrifugal filter unit with a 10 kDa membrane cutoff (Millipore). Concentrated V5-tagged PCSK9, its derivatives, Fc and Fcpro in conditioned media were quantitated by direct enzyme-linked immunosorbent assay detecting V5-tagged or Fc-proteins (R961-25, Invitrogen; AP113P, Millipore).

### Media transfer experiments

HepG2/shPCSK9 cells were seeded in a 12-well microplate at 3×10^5^ cells/well (Greiner Bio-One). After an overnight incubation, cells were washed and incubated in LPDS media (Dulbecco's phosphate-buffered saline (DPBS; Invitrogen), 0.1% sodium pyruvate (Invitrogen) and 5% lipoprotein-deficient serum (Biomedical Technologies Inc). Following 24 h incubation, media were replaced by conditioned media containing PCSK9 or its mutants at a final concentration of 1.5 µg/ml. After 4 h incubation at 37°C, cells were lysed in 1× RIPA buffer and then analyzed.

### Western blot analyses

Cells were recovered 48 h post-transfection, and lysed in 1× RIPA buffer (150 mM NaCl, 50 mM Tris-HCl, pH 8.0, containing 1% Nonidet P-40, 0.5% sodium deoxycholate, 0.1% SDS supplemented with 1× complete protease inhibitor mixture (Roche Applied Science)). Proteins in the cell lysates and media were resolved by 12% Tris- Glycine SDS-PAGE. The gels were blotted onto polyvinylidene difluoride (PVDF, Perkin Elmer Life Sciences) membranes (GE Healthcare), blocked for 1 h in TBS-T (50 mM Tris-HCl, pH 7.5, 150 mM NaCl, 0.1% Tween 20) containing 5% nonfat milk and immunoblotted with a homemade polyclonal human PCSK9 antibody (1∶1000) [12], human LDLR antibody (1∶1000, R&D Systems), beta-Actin (1∶5000; Sigma), monoclonal antibody (mAb) V5-HRP (1∶5000; Sigma), PCSK9 prosegment antibody (1∶1000; GenScript); Fc-HRP (1∶10000; Cedarlane Millipore). Appropriate horseradish peroxydase-conjugated secondary antibodies (1∶10000, Sigma) were used for detection with enhanced chemiluminescence using the ECL Plus kit (GE Healthcare). Quantification of protein bands was obtained using Image J software.

### Far western analyses

Two microgram of Fc-, Fcpro- or Fcpro QH-containing conditioned media were heated in reducing Laemmli sample buffer, resolved by SDS-PAGE on 10% tris-glycine gels, and electrotransfered onto polyvinylidene difluoride (PVDF, Perkin Elmer Life Sciences) membranes (GE Healthcare). Membranes were incubated for 1 h with 5% no fat milk in Tris-buffered saline, 0.1% Tween (TBST), the membranes where then incubated with conditioned medium produced in HepG2/shPCSK9 cells overexpressing either an empty vector (pIRES-V5), PCSK9-V5 or incubated with purified PCSK9 protein for 3 h at 4°C. The membranes were then washed in TBST and incubated with the polyclonal anti-human PCSK9 homemade antibody [12] or with an HRP conjugated anti-IgG1 Fc antibody. The bands were revealed by enhanced chemiluminescence (GE Healthcare).

## Results

### Expression and secretion of recombinant Fcpro protein

**Figure 1:**
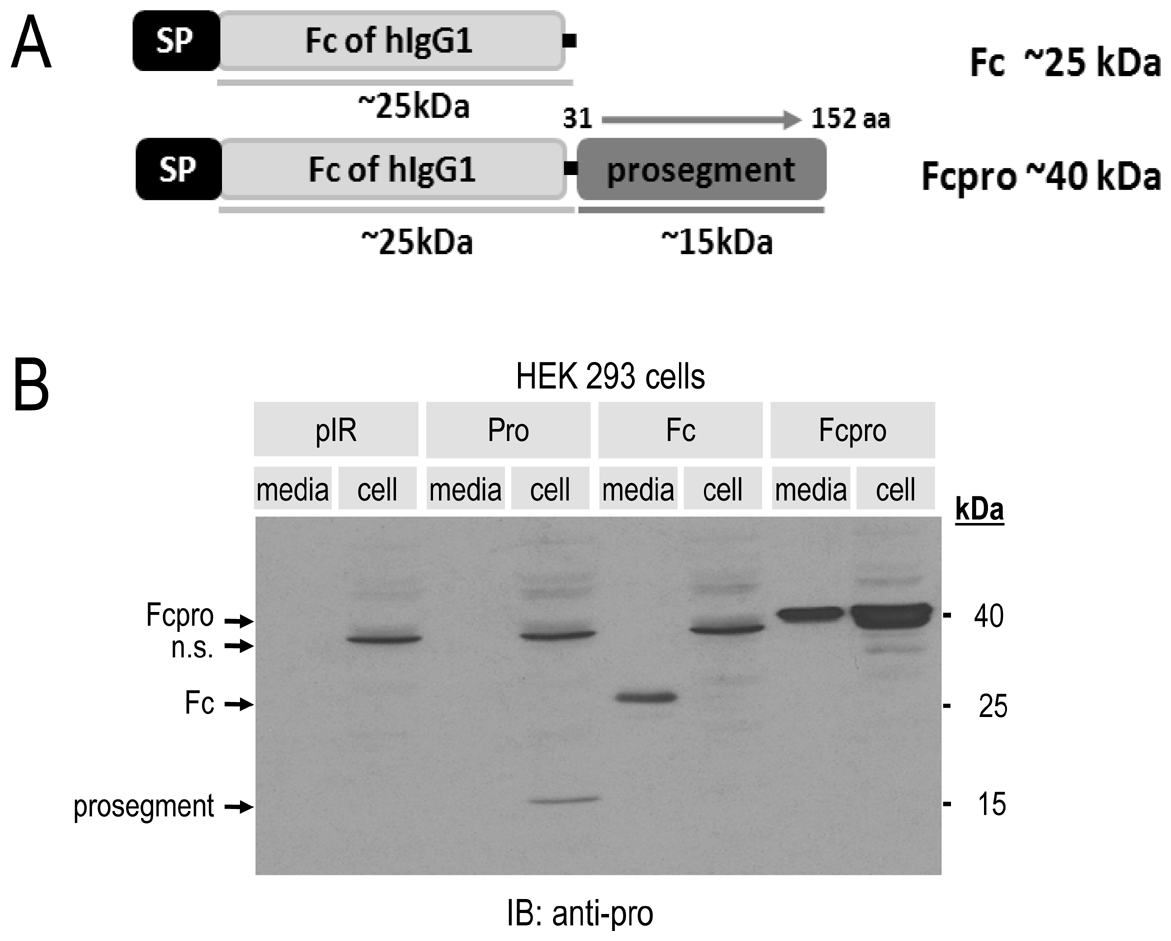
Expression and secretion of Fcpro chimera. **A**) Schematic representation of Fc and Fcpro constructs. *SP*: signal peptide. *Prosegment*: human PCSK9 aa 31 to 152. **B**) HEK293 cells transiently transfected with cDNAs encoding either *pIR*: control empty pIRES vector; *Pro*: PCSK9 prosegment; *Fc*: human IgG1 fragment crystallizable region; *Fcpro*: Fc fraction fused to PCSK9 prosegment. 48 h post-transfection, proteins in cell lysates and media were resolved by 12% SDS-PAGE, analyzed by Western blot and immunoblotted using a polyclonal anti-human PCSK9 prosegment antibody followed by horseradish peroxydase-conjugated secondary antibody.

Transient transfection of a cDNA coding for the prosegment alone in HEK293 or in HepG2 cells confirmed its inability to exit the cells, as previously observed [18]. We first attempted to produce a N-terminal His-tagged PCSK9 prosegment by cloning the cDNA coding for aa 31–152 into the pET16b vector and transforming the construct into the bacterial expression host BL21(DE3). After screening transformants, testing protein production, scaling-up and purification of the His-tagged PCSK9 prosegment, we end up with a very poor production level after dialysis and concentration of 10 L batches, with a total yield of ∼65 µg of pure protein/L (*not shown*). To circumvent this poor yield, we fused the Fc fragment of human immunoglobulin IgG1 with the N-terminus of the hPCSK9 prosegment (aa 31–152), resulting in an Fcpro chimera ending at natural C-terminus Gln_152_ of the prosegment (Figure 1A). Transient transfection of this recombinant Fcpro protein in HEK293 cells (Figure 1B) or HepG2/shPCSK9 cells (lacking endogenous PCSK9 [16]) resulted in a well expressed and secreted Fcpro protein. At 72 h post-transient transfection, we also measured the level of secreted Fcpro by direct-ELISA and found an average production yield of ∼4 µg/ml (∼4 mg/L). Upon incubation of the membrane with the secondary antibody recognizing Fc regions, we observed in the media a protein band corresponding to that of the control Fc protein alone (∼25 kDa), with much lower levels seen in cell lysates, likely due to an efficient secretion, as expected [19]. Interestingly, the Fcpro protein was also well secreted (∼40 kDa) but less so than the Fc alone, as a significant portion is retained in the cells (Figure 1B).

### Down-regulation of LDLR degradation by co-expression of PCSK9 and Fcpro recombinant protein

**Figure 2:**
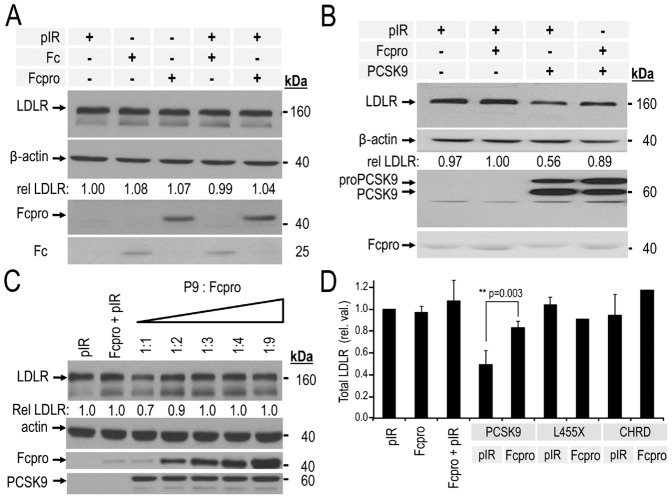
Intracellular *trans*-acting inhibitory effect of Fcpro on PCSK9 capacity to induce LDLR degradation. HepG2/shPCSK9 cells were co-transfected: **A**–**D**) with an empty pIRES vector (pIR) or human IgG1 fragment crystallizable region (Fc), both as negative controls; with the wild type human PCSK9; PCSK9 lacking CHRD (L455X); the CHRD alone, or the PCSK9 prosegment fused to Fc (Fcpro). **C**) Cells were co-transfected with wild type human PCSK9 and increasing ratios of Fcpro. Cell lysates were resolved by 12% SDS-PAGE and proteins analyzed by Western blot. **B**,**D**) Total LDLR was detected using a polyclonal anti-human LDLR and its levels were normalized relative to β-actin cellular loading controls. Fc and Fcpro proteins were detected using a polyclonal anti-Fc antibody. PCSK9, L455X and CHRD proteins were detected using a monoclonal anti-V5 antibody. Statistical values were estimated by Student's t-test and considered significant when p-values were <0.05. The data show that co-expression of Fcpro with PCSK9 significantly reduces its capacity to induce LDLR degradation (**p = 0.003). These data are representative of at least three independent experiments.

Since PCSK9 is mostly expressed in hepatocytes [3], which are the main source of circulating pPCSK9 [20], [21], we decided to test our constructs in these cells. We first tested the effect of Fcpro on naïve HepG2 cells and showed that its transient overexpression led to a ∼1.6 fold increased levels of total LDLR (Figure S1A), suggesting that it can inhibit the effect of endogenous PCSK9 in these cells. However, it was also pertinent to choose a cell type devoid of endogenous PCSK9. Thus, we used the previously reported human hepatocyte-derived HepG2 cell line lacking PCSK9 expression by stable shRNA silencing (HepG2/shPCSK9 cells) and that have been tested to be even more sensitive to the PCSK9-induced degradation of their endogenous LDLR as compared to wild type HepG2 cells [9], [16], [18]. We also performed experiments in the widely used human embryonic kidney epithelial HEK293 cells, because they do not express PCSK9, are sensitive to exogenous PCSK9-induced degradation of the LDLR [16], and exhibit a better transfection efficiency than HepG2 cells. To induce a higher expression of LDLR, HEK293 cells were first grown for 24 h in a lipoprotein deficient serum (LPDS) for 24 h before transfection. We first tested the effect of the control protein Fc or the Fcpro recombinant protein on both cell types, lacking endogenous PCSK9. Western blot analysis of the HepG2/shPCSK9 cell lysates (Figure 2A), or the HEK293 cells (Figure S1B) recovered 48 h post-transfection of the empty pIRES vector, Fc or Fcpro revealed no effect on total LDLR cellular protein levels, showing that the use of the empty vector or the Fc alone constitute good negative controls for the ensuing experiments. It should be emphasized that Fcpro alone does not affect LDLR levels in two cell types that lack endogenous PCSK9. In order to test the inhibitory effect of our recombinant Fcpro in HEK293 cells, we co-expressed it with PCSK9 at a DNA ratio of PCSK9:Fcpro of 1∶3, a ratio usually used for other convertases [22]. Western blot analysis of cell lysates revealed that the ∼44% enhanced LDLR degradation by PCSK9, is ∼75% reversed when Fcpro is co-expressed (only 11% reduction in LDLR levels left) (Figure 2B). In order to test whether the transfection ratio was optimal, we increased the ratio in favor of Fcpro, up to 9-fold, keeping PCSK9 constant. The data showed that maximum inhibitory activity of Fcpro was already achieved at the 1∶3 ratio (Figure 2C), prompting us to keep this ratio in further experiments. In Figures 2D (HepG2/shPCSK9 cells) and S1 (HEK293 cells), we tested the effect of the co-expression of Fcpro with full length PCSK9 or with its domain-deleted forms. These included PCSK9 lacking the CHRD domain (L455X) or the C-terminal Cys-His-rich domain (CHRD) alone [12]. The latter constructs do not have any effect on total LDLR levels [18] and thus served as controls. The results showed that only full length PCSK9 activity is inhibited by Fcpro, and that the latter has no significant effect on LDLR in the presence or absence of the domain-deleted constructs.

**Figure 3:**
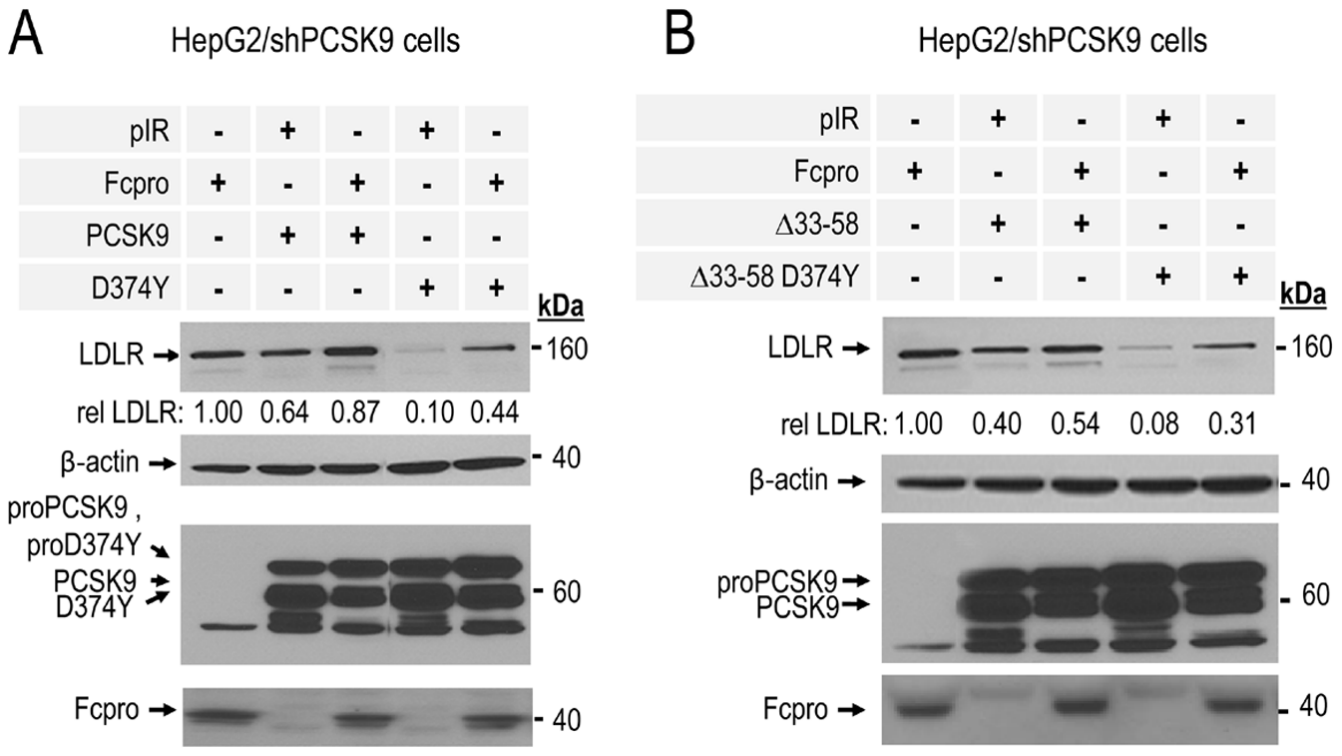
LDLR degradation induced by PCSK9 GOF mutants is decreased by their co-expression with Fcpro. HepG2/shPCSK9 cells were co-transfected with **A**) and **B**) pIR empty vector as a negative control or PCSK9 prosegment fused to the Fc region chimera (Fcpro) with **A**) wild type human PCSK9 or its GOF natural mutant PCSK9 D374Y (D374Y); **B**) PCSK9 GOF mutants: prosegment acidic stretch deletant Δ33-58 (Δ33-58) or Δ33-58 D374Y. Cells lysates were resolved by 10% SDS-PAGE and proteins analyzed by Western blot. Total LDLR was detected using a polyclonal anti-human LDLR and its levels were normalized relative to β-actin cellular loading controls. Fcpro proteins were detected using a polyclonal anti-Fc antibody. PCSK9 or its GOF mutant proteins were detected using a monoclonal anti-V5 antibody. These data are representative of at least three independent experiments.

We next sought to compare the inhibitory effect of Fcpro on the activity of PCSK9 and that of the most powerful D374Y GOF mutant as well as that of a GOF construct consisting of either the wild type sequence or the D374Y mutant both lacking the acidic N-terminal region of the prosegment (Δ33-58) [18]. Thus, Western blot analyses of cell lysates of HepG2/shPCSK9 cells co-expressing of Fcpro with these constructs revealed that Fcpro is also capable of decreasing their activity to induce cellular LDLR degradation. When compared with wild type PCSK9 or its Δ33-58 derivative, it seems that the average 4-fold increased LDLR levels in the presence of Fcpro and the GOF mutants (D374Y or Δ33-58 D374Y) as compared to the GOF D374Y or Δ33-58 D374Y alone (0.1, 0.08 *versus* 0.44, 0.31) is more efficient that the ∼1.4-fold effect of Fcpro in the presence of WT or Δ33-58 PCSK9 (Figures 3A,B).

### Fcpro inhibits LDLR degradation induced by extracellular PCSK9

**Figure 4:**
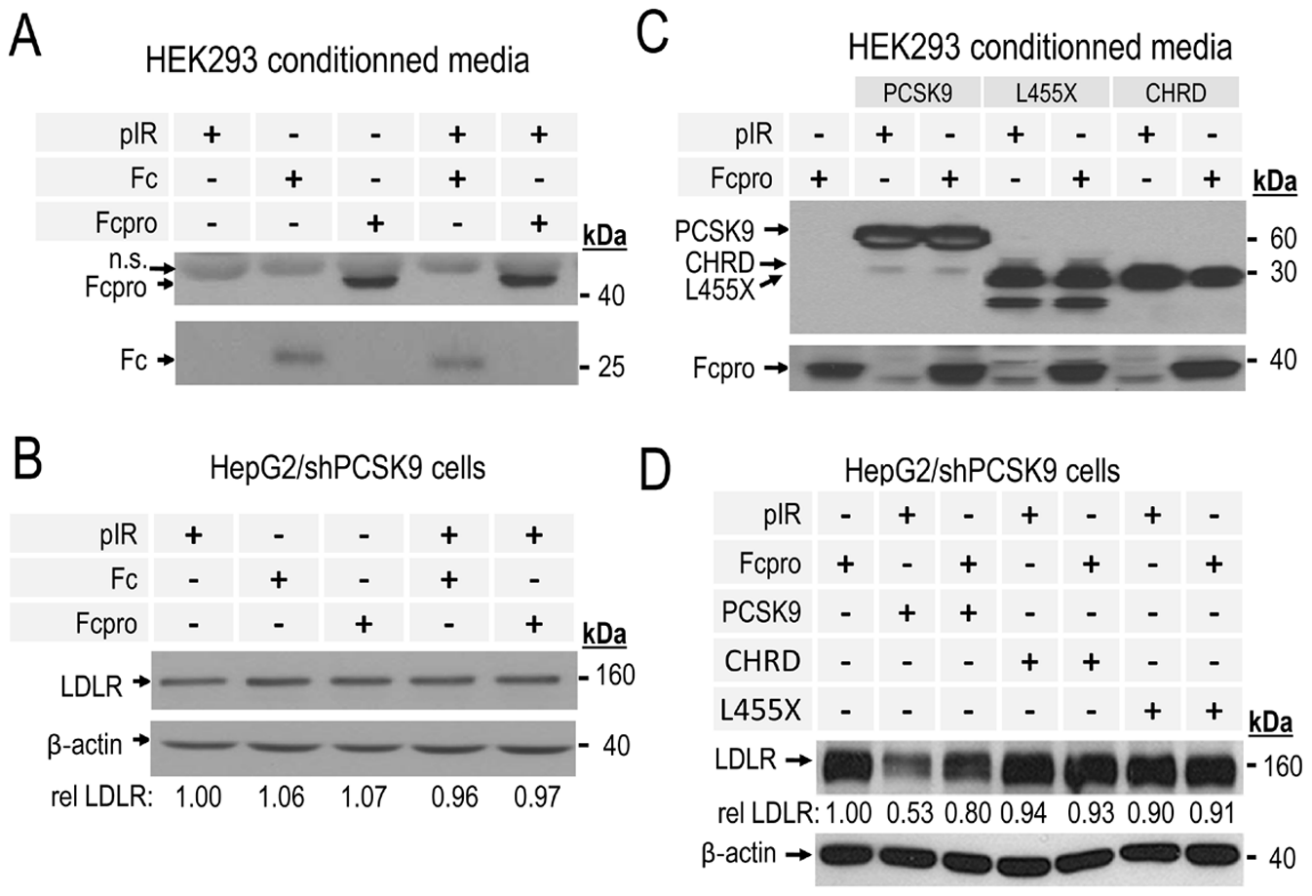
Extracellular *trans*-acting inhibitory effect of Fcpro co-incubated with PCSK9 on LDLR degradation. **A**) and **C**) Secreted Fc (human IgG1 fragment crystallizable region), Fcpro (Fc fused to PCSK9 prosegment), human wild type PCSK9 or its deletants forms L455X or CHRD proteins contained in conditioned media from transiently transfected HEK293 cells were analyzed following 10% SDS-PAGE by Western blot. Fc and Fcpro proteins were detected using a polyclonal anti-Fc antibody. n.s. indicate a non-specific band. PCSK9, L455X and CHRD proteins were detected using a mAb anti-V5. **B**) and **D**) HepG2/shPCSK9 cells were incubated with 1∶3 (weight∶weight) ratio of PCSK9 or L455X or CHRD to Fc or Fcpro amounts of proteins (quantified by ELISA assays) for 4 h at 37°C. Cells were then lysed, and LDLR levels were analyzed by Western blot analysis and normalized relative to β-actin cellular loading controls. Total LDLR was detected using a polyclonal antibody anti-human LDLR and its levels were normalized relative to β-actin cellular loading controls. These data are representative of at least three independent experiments. **A**–**D**) pIR: control empty pIRES vector.

Based upon the ability of secreted PCSK9 to bind directly to the extracellular domain of LDLR, we tested if the recombinant Fcpro protein co-incubated with an extracellular PCSK9 would compromise its function, and thus prevent cellular LDLR degradation by this route. Accordingly, we concentrated ∼10× the media produced in HEK293 cells obtained after 72 h post-transfection with each cDNA construct. The concentration of each V5-tagged or Fc region containing protein in these media was assessed by ELISA-V5 or ELISA-Fc. We first verified the potential extracellular activity of Fc or Fcpro alone on the human cell line HepG2/shPCSK9. The recombinant proteins obtained from conditioned media of HEK293 cells and used for the extracellular effect experiments are shown in Figure 4A. The media were adjusted to a final concentration of 3 µg/ml of each protein before the 4 h cell incubations. Western blot analysis of the cell lysates are shown in Figure 4B where the levels of total cellular LDLR are very similar, revealing the absence of activity of the Fc- or Fcpro-containing conditioned media after an incubation period of 4 h at 37°C with HepG2/shPCSK9 cells. Thus, Fc or Fcpro proteins containing media represent negative controls for further extracellular-effect experiments. Next, we pre-incubated conditioned media containing 3 µg/ml of Fc or Fcpro with 1 µg/ml of full length PCSK9 or its domain deletants (L455X or CHRD) (protein ratio 1∶3). HepG2/shPCSK9 cells were then incubated with these pre-incubated conditioned media (Figure 4C) under the same conditions as in Figure 4B. Western blot analysis of total LDLR in these cells lysates (Figure 4D) showed a decrease of ∼50% of LDLR cellular levels induced by extracellular PCSK9 incubation. However, in the presence of Fcpro this effect was significantly reduced by ∼60% (from ∼50% to ∼20% total LDLR degradation). Moreover, as seen in intracellular overexpression experiments, extracellular L455X or the CHRD did not affect LDLR levels in the presence or absence of Fcpro.

### Evidence for direct binding of Fcpro to PCSK9

**Figure 5:**
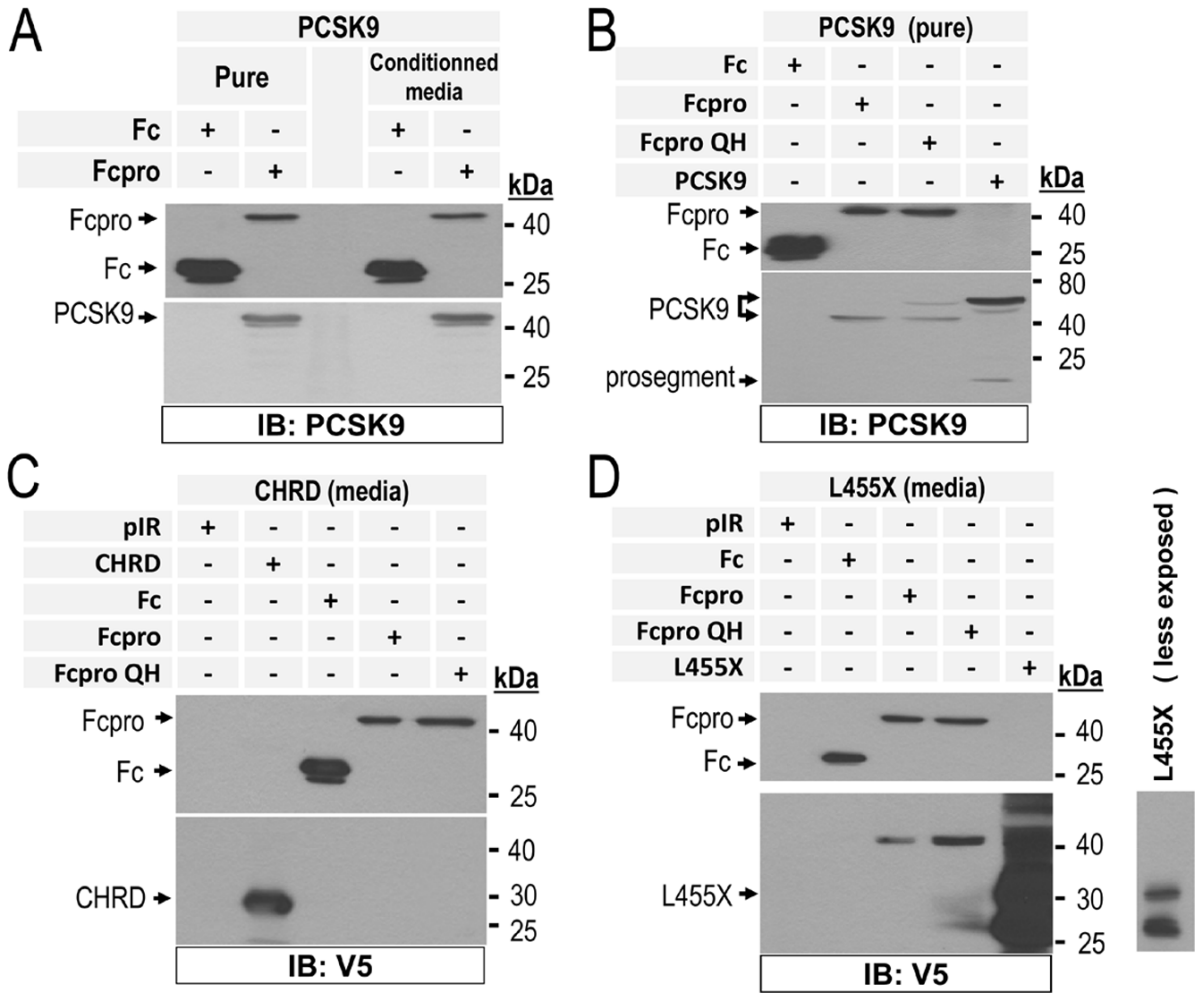
*In vitro* association of PCSK9 and Fcpro chimera. Direct binding of Fcpro to PCSK9 was analyzed by Far Western blot. Concentrated conditioned media from HEK293 cells containing 2 µg of Fc or Fcpro or Fcpro QH proteins were resolved by 12% SDS-PAGE. Proteins were transferred onto a PVDF membrane and were incubated 4 h with: **A**) (right panel) and **B**) 15 µg/mL of purified human wild type PCSK9 (quantified by ELISA assays) and in A) (left panel), **C**) and **D**) with conditioned media from HepG2/shPCSK9 cells containing 5 µg/mL of V5-tagged wild type PCSK9 or its domain deletants L455X or CHRD proteins. **A**) and **B**) binding of PCSK9 to proteins bound on the PVDF membranes were detected by using our polyclonal homemade anti-human PCSK9 antibody. **C**) and **D**) Binding of the PCSK9 domain deletants variants were detected by using horseradish peroxydase conjugated-V5 mAb. These data are representative of at least three independent experiments.

To assess if the interfering activity of Fcpro was due to a direct or indirect binding to PCSK9, we used a Far-Western based approach to detect protein-protein interactions *in vitro*. As prey proteins, we resolved 2 µg of Fc- or Fcpro-containing conditioned media on an SDS-PAGE 10% tris-glycine gel and transferred the proteins onto a PVDF membrane. Next, the bait proteins consisted of either human purified recombinant PCSK9 or conditioned media containing PCSK9. Following 4 h incubations, extensive washes were performed to remove all unbound protein from the surface of the PVDF membrane. Immunodetection using an anti-human PCSK9 antibody [12] revealed the presence of PCSK9 bound to Fcpro protein (∼40 kDa) but not to Fc control protein (∼25 kDa) (Figure 5A). Thus, since the result was the same with either pure PCSK9 or the one derived from conditioned media, this eliminated the possibility of another protein in the conditioned media could mediate the Fcpro≡pPCSK9 interaction. This result supports the notion that Fcpro directly binds pPCSK9.

We recently showed that Gln_152_ represents a critical residue for PCSK9 recognition and autocatalytic cleavage, as only its replacement by Met or Ala marginally reduced cleavage [23]. Furthermore, the LOF Q152H natural mutant [24] is not processed and acts a dominant negative resulting in a significant reduction of zymogen processing, thereby abrogating PCSK9 secretion [23], [24]. Thus, we tested the direct binding of PCSK9 to either Fcpro or its single point mutant with the C-terminal Gln substituted by His (Fcpro QH). The data show that both proteins directly bind pure pPCSK9 (Figure 5B) or PCSK9 obtained from conditioned media (*not shown*). This reveals that the C-terminal Gln in Fcpro is not critical for such interaction, nor does it affect the inhibitory activity of Fcpro or Fcpro QH on PCSK9-induced LDLR degradation (Figure S2), yet it is critical for productive binding to the catalytic pocket of PCSK9 [23].

We thus considered the possibility that Fcpro QH may bind another region of pPCSK9. This hypothesis is further supported by our earlier observation that the prosegment and its Q152H as well as Q152W mutants can all bind the zymogen in the ER, as well as PCSK9-Δpro that lacks its prosegment. This was also seen using Fcpro and Fcpro QH (Figure S3). However, only the wild type prosegment can productively interact with PCSK9-Δpro and allow its secretion [23].

To dissect which structural part of PCSK9 could be implicated in the binding to Fcpro, we repeated the Far Western experiment using conditioned media containing 2 µg of either CHRD or L455X proteins (Figures 5C, D). Interestingly, only the L455X protein bound Fcpro, but not the CHRD. This showed that Fcpro binds the prosegment and/or the catalytic domain of PCSK9.

Finally, we tested the possibility that Fcpro could bind the prosegment of pPCSK9. For this we generated a pPCSK9 in which the prosegment is V5-tagged at the N-terminus (Figure S4A), by co-expression V5-prosemgent with PCSK9Δpro [23]. Unexpectedly, while Fcpro bound the prosegment≡PCSK9 (Figure 5B), it did not bind the V5-prosegment≡PCSK9 (Figure S4B), suggesting that the V5-tag inserted at the N-terminus of the prosegment blocks the binding of pPCSK9 to Fcpro.

## Discussion

Since the validation by genetic studies that PCSK9 has a clear role in the regulation of cholesterol homeostasis, many efforts have been made to develop an inhibitor of this attractive therapeutic target for the treatment of hypercholesterolemia [1]. Considering that PC-prosegments may represent important molecules that regulate enzymatic activity, some of them were previously used as potent inhibitors against their cognate convertases [22], [25]–[27]. We previously demonstrated that the removal of the acidic stretch of the PCSK9 prosegment resulted in not only a higher binding but also in an increased activity of the convertase on LDLR [18]. Based on these observations, we hypothesized that while acting as a “natural brake” for the enzymatic activity of PCSK9, the prosegment could also inhibit its non-enzymatic activity on specific targets such as LDLR, and thus act as a “dominant negative molecule” interfering with the mechanism of LDLR degradation induced by PCSK9.

Our previous efforts to produce a PCSK9 prosegment in bacterial cultures resulted in a poor yield of this fragment. In this work, we overcome this obstacle by generating a recombinant fusion protein of the Fc region from human immunoglobulin IgG1 to the prosegment of human PCSK9 (Fcpro) (Figure 1A). This strategy allowed the prosegment to be successfully expressed and secreted (Figure 1B) in a yield that was ∼62-fold higher than bacteria. The advantage of using the Fc portion of the immunoglobulin IgG1 greatly facilitated the expression and secretion of the recombinant protein. Moreover, this extension can also provide additional advantages as proteins fused to Fc regions have improved solubility and stability and can be produced and purified in a large scale using a protein A affinity chromatography [28]. Indeed, most of the successful fusion protein therapeutic approaches today contain different Fc-proteins of immunoglobulins [29].

Herein, we demonstrate for the first time that a chimera containing the prosegment (Fcpro) directly binds to pPCSK9 (Figure 5) and effectively acts as a negative regulator of its ability to induce LDLR degradation. This direct down-regulation of pPCSK9 activity was revealed by intracellular co-expression experiments of Fcpro with either wild type PCSK9 or two of its GOF mutants. The data showed that all PCSK9 forms exhibited reduced activity on LDLR in the presence of Fcpro (Figures 2,3). This inhibitory effect was also observed to be valid extracellularly, whereupon pre-incubation of pPCSK9 with Fcpro resulted in almost total inactivation of the pPCSK9 ability to induce the degradation of cellular LDLR (Figure 4).

Concerning the pPCSK9 region that binds Fcpro, our data suggested that it could implicate the prosegment following the acidic stretch (i.e., aa 59–152), excluding the C-terminal Gln_152_ (Figure S2). Indeed, earlier data showed that the zymogen proPCSK9 can oligomerize in the ER and that such oligomers can be dissociated using a reducing agent [3]. Subsequent studies revealed that the prosegment alone can also oligomerize in the ER [23]. We further deduce that the acidic region of the prosegment (aa 33–58) is not implicated, since the PCSK9 Δ33-58 is still inhibited by Fcpro (Figure 3B). Since the bulky Trp_152_ is not expected to productively enter the tight catalytic pocket of PCSK9, yet the prosegment Q152W still binds the zymogen [23], this strongly suggests that the postulated second binding region seemingly does not implicate the catalytic pocket *per se*, but may be due to either a prosegment≡prosegment and/or prosegment≡catalytic domain interaction. However, we cannot exclude the possibility that the bulky Fcpro could also modify the catalytic subunit either by direct binding or due to steric hindrance. In that context, overexpression of the prosegment alone with full length PCSK9 resulted in a significant decrease in level of the furin-cleaved form at Arg_218_↓ [23], revealing that the *in trans* binding of the overexpressed prosegment allosterically modifies the catalytic subunit in such as way that the cleavage of PCSK9 by furin is largely restricted.

Since PCSK9 is now considered a major target for lowering high levels of circulating LDL-cholesterol, which is highly atherogenic and can lead to cardiovascular failure, a number of pharmaceutical companies are developing potent inhibitors of circulating PCSK9 that would prevent its ability to enhance the degradation of liver LDLR. The most promising present strategies to inhibit PCSK9 includes the use of blocking monoclonal antibodies (mAb) or fibronectin fragments (adnectins) that prevent the formation of the pPCSK9≡LDLR complex at the cell surface [1], [30]. Recently, mAbs against PCSK9 that block its interaction with the LDLR have clearly shown very promising results and are now in Phase-II and -III clinical trials [1], [31]. However, such conventional antibodies possess many intrinsic negative characteristics as drugs. In general, they are high-molecular mass proteins (∼150 kDa), complex to manufacture, and potentially immunogenic; they are unsuited to oral delivery. Above all, the high cost associated with the development and manufacture of mAbs limits their wide applicability to all but advanced stages of serious diseases.

The present work presents an alternative, new strategy to develop PCSK9 inhibitors by interfering with the structure of pPCSK9 and exploiting the properties of the PCSK9 prosegment and the advantage of its fusion to a humanized Fc of IgG1. This fusion strategy has been very successful in the treatment of various diseases by administration of specific Fc-chimeras, including rheumatoid arthritis and other autoimmune diseases [29]. The proposed Fcpro fusion protein could be injected to knockin mice expressing human PCSK9 at the place of mouse PCSK9 in order to further test its potency on LDL lowering *in vivo*. Once validated and toxicity and pharmacodynamic studies performed this Fcpro inhibitor could then conceivably move to preclinical studies, as was done for mAbs [1], [30]. Furthermore, in the future modifications of Fc to create smaller monomeric forms of Fcpro [32] could also be made to enhance the bio-availability of this construct towards its target pPCSK9. In conclusion, the present mAbs results are very encouraging and may well lead to a novel approach to combat hypercholesterolemia and possibly other PCSK9-related pathologies [33]. The alternative use of Fcpro as a potential PCSK9 inhibitor offers the advantage of compounds easier to manufacture at lower cost. The future will tell whether this approach is more attractive than the presently used mAb strategy.

## Supporting Information

Figure S1 **Decreased PCSK9-mediated degradation of LDLR in transiently transfected HepG2 cells with Fcpro and co-transfected HEK293 cells with PCSK9 and Fcpro.** **A**) Naïve HepG2 cells were transfected with either an empty pIRES vector (pIR) or with the PCSK9 prosegment fused to the Fc region chimera (Fcpro), and **B**) HEK293 cells were co-transfected with an empty pIRES vector (pIR) or Fcpro and the wild type human PCSK9, or PCSK9 lacking the CHRD (L455X), or the CHRD alone. Cells lysates were resolved by 12% SDS-PAGE and proteins analyzed by Western blot. Total LDLR was detected using a polyclonal anti-human LDLR and its levels were normalized relative to β-actin cellular loading controls. Fcpro proteins were detected using a polyclonal anti-Fc antibody. PCSK9, L455X and CHRD proteins were detected using a monoclonal horseradish peroxydase conjugated-V5 antibody. These data are representative of at least three independent experiments.
(TIF)

Figure S2 **Similar inhibitory effect on LDLR degradation induced by PCSK9 when co-expressed with Fcpro or Fcpro QH.** Cell lysates of HEK293 cells co-expressing Fcpro or Fcpro QH with human wild type PCSK9 were resolved by 10% SDS-PAGE. Total LDLR levels were analyzed by Western blot and LDLR proteins were detected by using a polyclonal anti-human LDLR antibody and its levels were normalized relative to β-actin cellular loading controls. pIR: control empty pIRES vector.
(TIF)

Figure S3 **Pull-down of PCSK9 by Fcpro or Fcpro QH chimeras in HepG2/shPCSK9 cells.** Cell lysates of HepG2/shPCSK9 cells co-expressing Fc or Fcpro or Fcpro QH with a full length PCSK9 or a PCSK9Δprosegment were immunoprecipitated (IP) with an anti-PCSK9 prosegment antibody and the immunoprecipitates were resolved by 12% SDS-PAGE and analyzed by Western blot using a monoclonal horseradish peroxydase conjugated-V5 antibody (IB:V5). The migration positions of the zymogen of PCSK9 (proPCSK9) and PCSK9 are shown. This figure is representative of at least two independent experiments. pIR: control empty pIRES vector.
(TIF)

Figure S4 **Loss of Fcpro binding capacity to a N-terminally V5-tagged PCSK9 prosegment.** Concentrated conditioned media from HEK293 cells containing 2 µg of Fc or Fcpro or Fcpro QH proteins were resolved by 12% SDS-PAGE. Proteins were transferred onto a PVDF membrane and were incubated 4 h with conditioned media produced in HepG2/shPCSK9 cells (expressing *in trans* a N-terminus V5-tagged PCSK9 prosegment and a no-tagged PCSK9Δprosegment) containing 5 µg/mL of N-terminally V5-pro-PCSK9. Binding of N-tagged PCSK9 to proteins bound on the PVDF membrane was detected by using our polyclonal homemade anti-human PCSK9 antibody. These data are representative of at least three independent experiments. B) pIR: control empty pIRES vector.
(TIF)
