# Supplementary figures and images for: PCSK9 Prosegment Chimera as Novel Inhibitors of LDLR Degradation
Source: PLoS One. 2013 Aug 12;8(8):e72113. doi: 10.1371/journal.pone.0072113 (PMC3741231; doi:10.1371/journal.pone.0072113)

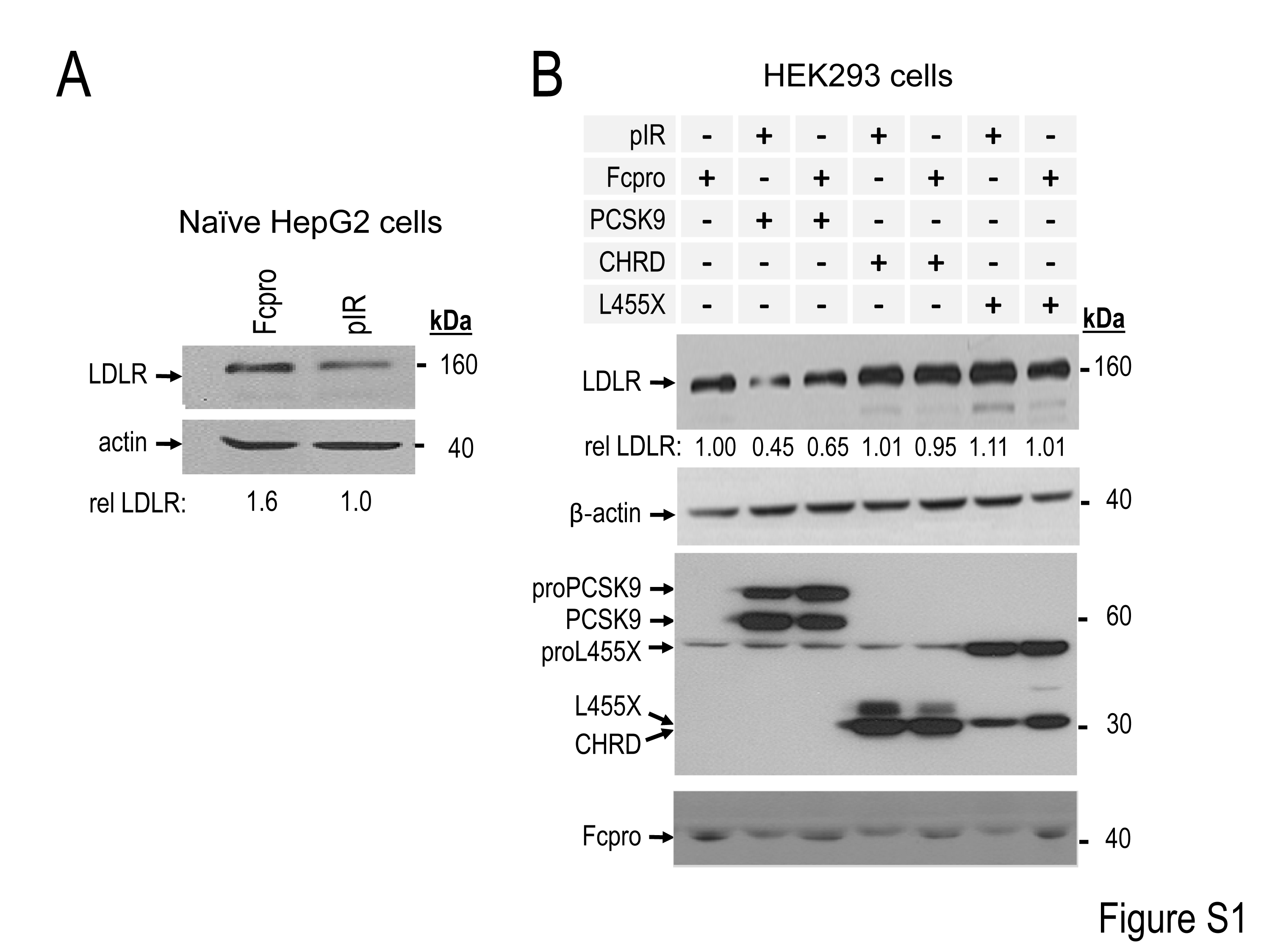

Supplement: Figure S1 — Decreased PCSK9-mediated degradation of LDLR in transiently transfected HepG2 cells with Fcpro and co-transfected HEK293 cells with PCSK9 and Fcpro. A) Naïve HepG2 cells were transfected with either an empty pIRES vector (pIR) or with the PCSK9 prosegment fused to the Fc region chimera (Fcpro), and B) HEK293 cells were co-transfected with an empty pIRES vector (pIR) or Fcpro and the wild type human PCSK9, or PCSK9 lacking the CHRD (L455X), or the CHRD alone. Cells lysates were resolved by 12% SDS-PAGE and proteins analyzed by Western blot. Total LDLR was detected using a polyclonal anti-human LDLR and its levels were normalized relative to β-actin cellular loading controls. Fcpro proteins were detected using a polyclonal anti-Fc antibody. PCSK9, L455X and CHRD proteins were detected using a monoclonal horseradish peroxydase conjugated-V5 antibody. These data are representative of at least three independent experiments. (TIF) [file pone.0072113.s001.tif]

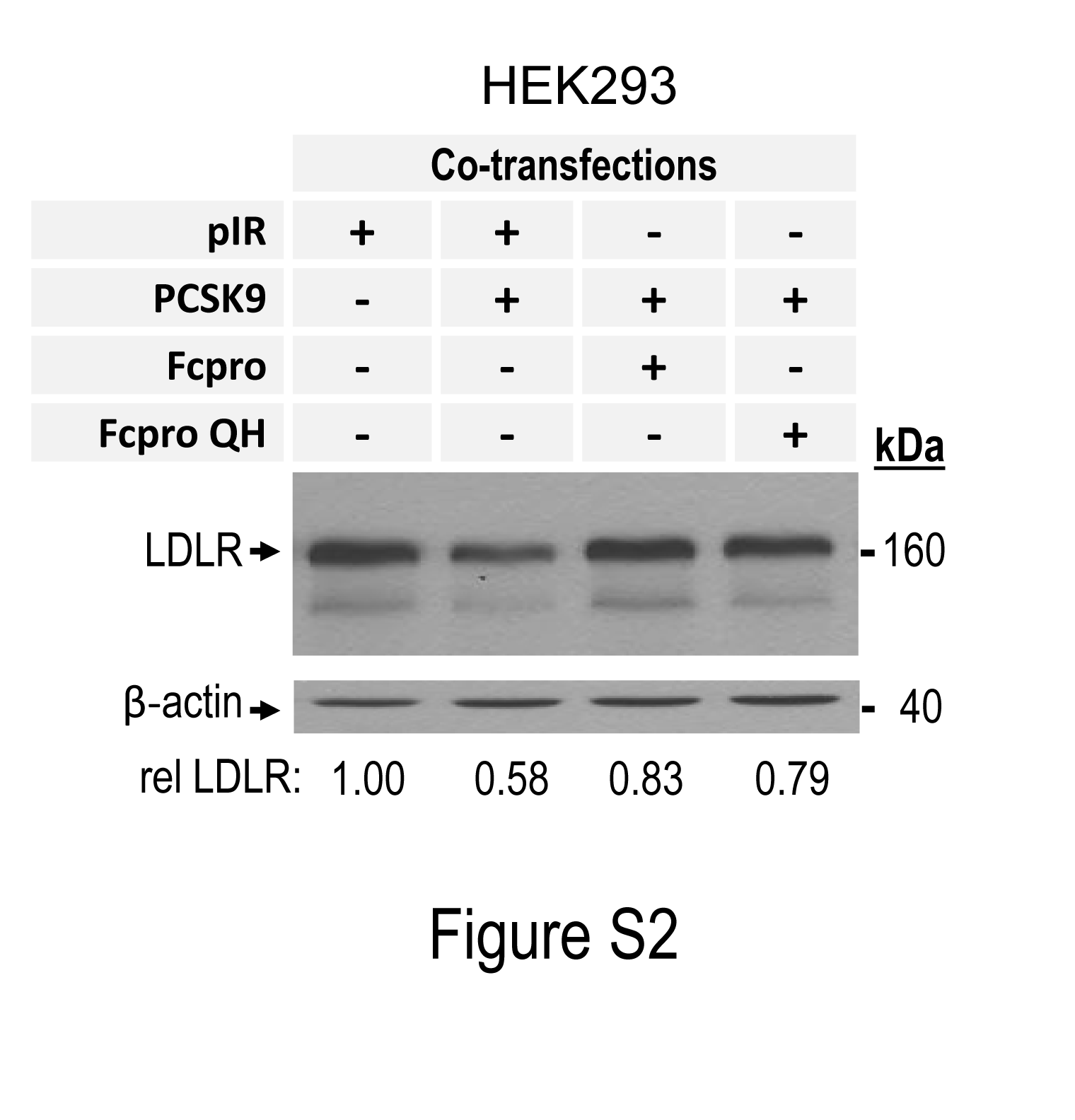

Supplement: Figure S2 — Similar inhibitory effect on LDLR degradation induced by PCSK9 when co-expressed with Fcpro or Fcpro QH. Cell lysates of HEK293 cells co-expressing Fcpro or Fcpro QH with human wild type PCSK9 were resolved by 10% SDS-PAGE. Total LDLR levels were analyzed by Western blot and LDLR proteins were detected by using a polyclonal anti-human LDLR antibody and its levels were normalized relative to β-actin cellular loading controls. pIR: control empty pIRES vector. (TIF) [file pone.0072113.s002.tif]

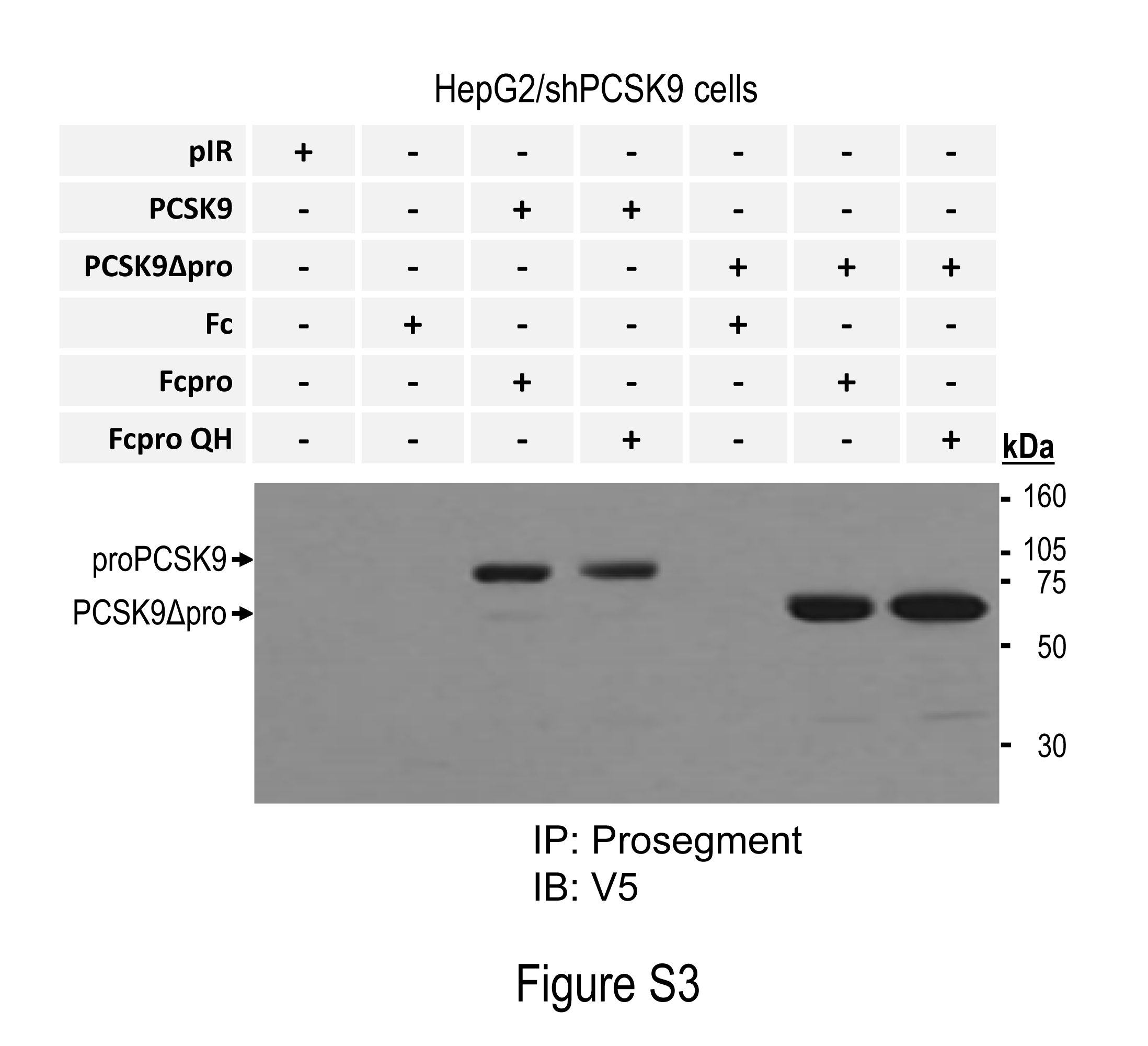

Supplement: Figure S3 — Pull-down of PCSK9 by Fcpro or Fcpro QH chimeras in HepG2/shPCSK9 cells. Cell lysates of HepG2/shPCSK9 cells co-expressing Fc or Fcpro or Fcpro QH with a full length PCSK9 or a PCSK9Δprosegment were immunoprecipitated (IP) with an anti-PCSK9 prosegment antibody and the immunoprecipitates were resolved by 12% SDS-PAGE and analyzed by Western blot using a monoclonal horseradish peroxydase conjugated-V5 antibody (IB:V5). The migration positions of the zymogen of PCSK9 (proPCSK9) and PCSK9 are shown. This figure is representative of at least two independent experiments. pIR: control empty pIRES vector. (TIF) [file pone.0072113.s003.tif]

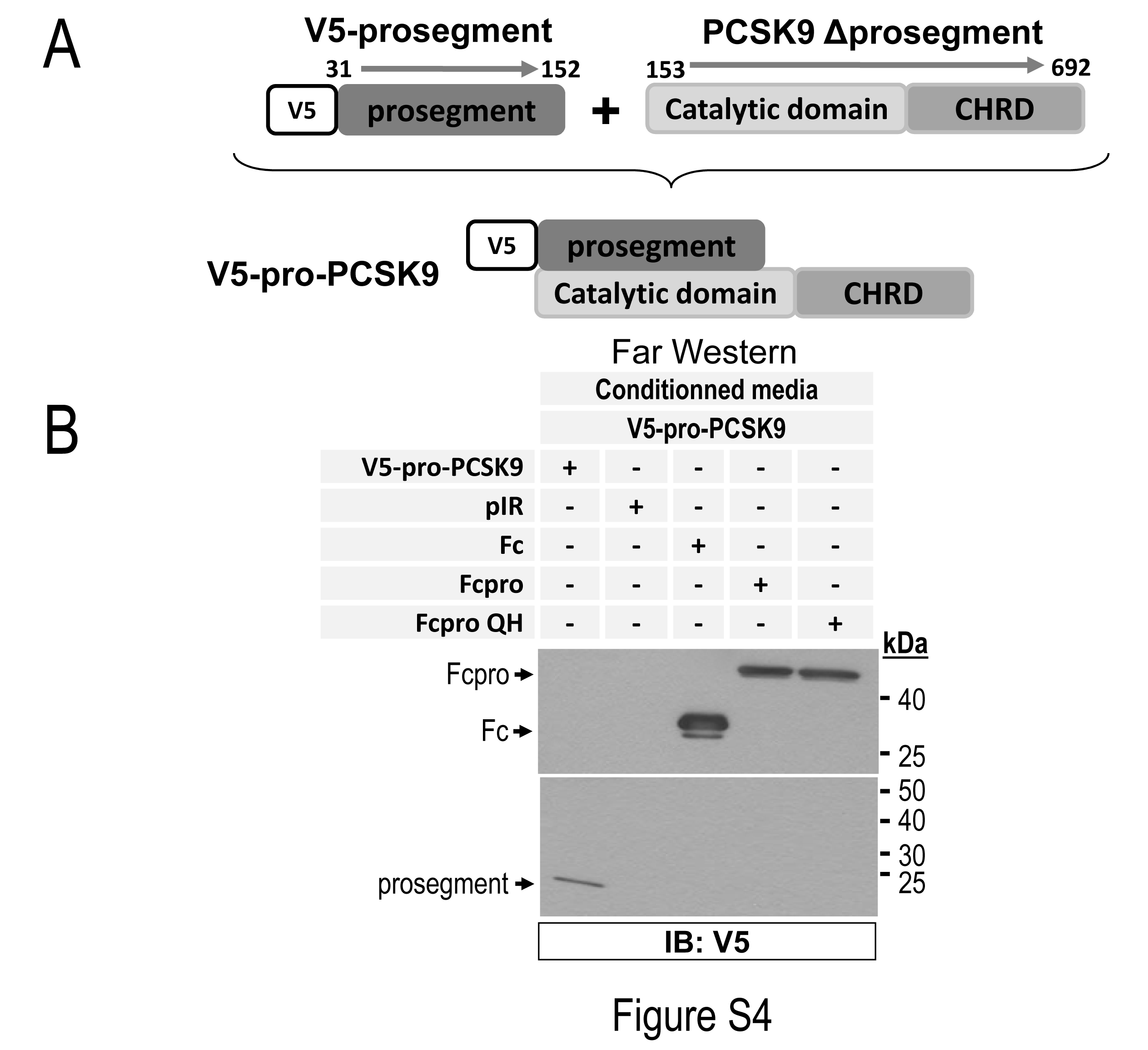

Supplement: Figure S4 — Loss of Fcpro binding capacity to a N-terminally V5-tagged PCSK9 prosegment. Concentrated conditioned media from HEK293 cells containing 2 µg of Fc or Fcpro or Fcpro QH proteins were resolved by 12% SDS-PAGE. Proteins were transferred onto a PVDF membrane and were incubated 4 h with conditioned media produced in HepG2/shPCSK9 cells (expressing in trans a N-terminus V5-tagged PCSK9 prosegment and a no-tagged PCSK9Δprosegment) containing 5 µg/mL of N-terminally V5-pro-PCSK9. Binding of N-tagged PCSK9 to proteins bound on the PVDF membrane was detected by using our polyclonal homemade anti-human PCSK9 antibody. These data are representative of at least three independent experiments. B) pIR: control empty pIRES vector. (TIF) [file pone.0072113.s004.tif]
